# Malocclusions and quality of life among adolescents: a systematic review and meta-analysis

**DOI:** 10.1093/ejo/cjad009

**Published:** 2023-03-30

**Authors:** Emma Göranson, Mikael Sonesson, Aron Naimi-Akbar, Lillemor Dimberg

**Affiliations:** Center for Orthodontics and Pediatric Dentistry, Norrköping, Public Dental Service Östergötland, Norrköping, Sweden; Department of Biomedical and Clinical Sciences, Linköping University, Linköping, Sweden; Department of Orthodontics, Malmö University, Malmö, Sweden; Department of Orthodontics, Malmö University, Malmö, Sweden; HTA-O Health Technology in Odontology, Malmö University, Malmö, Sweden; Department of Orthodontics, Malmö University, Malmö, Sweden; HTA-O Health Technology in Odontology, Malmö University, Malmö, Sweden; Department of Orthodontics, Folktandvården Stockholms län AB, Folktandvården Eastmaninstitutet, Stockholm, Sweden

## Abstract

**Background:**

Malocclusions in adolescents might have a negative impact on oral health-related quality of life (OHRQoL). Potential confounding variables (confounders) such as age, gender, caries, and socioeconomic status may skew the real relationship between malocclusions and OHRQoL.

**Objectives:**

To analyse the effect of malocclusions in adolescents on OHRQoL, when controlled for potential confounders.

**Search methods:**

Five databases (PubMed, Cochrane Library, Cinahl, Scopus, and Web of Science) were searched up to 15 June 2022.

**Selection criteria:**

Studies in which OHRQoL in 10–19-year olds with and without malocclusions were compared.

**Data collection and analysis:**

Screening, data extraction, and quality assessments were performed by four investigators independently. Risk of bias was assessed according to the Swedish Agency for Health Technology Assessment and Assessment of Social Services (SBU) guidelines. To be included, studies had to control for confounders. Certainty of evidence was assessed with GRADE.

**Results:**

Thirteen cross-sectional studies with low and moderate risk of bias were included in the qualitative synthesis. Four of these were also included in the quantitative synthesis (meta-analysis). The 13 studies in the qualitative synthesis displayed a large variation among the indices used for malocclusion ratings, as well as in instruments measuring OHRQoL. There was moderate quality of evidence that malocclusions have a negative effect on OHRQoL. The four articles included in the quantitative synthesis (meta-analysis) measured malocclusions with DAI and OHRQoL with CPQ 11–14 short form. There was moderate quality of evidence that malocclusions have a negative effect on OHRQoL (RR/PR 1.15, 95% CI 1.12–1.18, 3672 participants).

**Conclusions:**

There is moderate quality of evidence that malocclusions in adolescents have a negative impact on OHRQoL, after taking relevant confounders into consideration. Future studies should ideally use standardized measures for malocclusion ratings and OHRQoL.

**Registration:**

PROSPERO. CRD42020186152.

## Introduction

Malocclusions are highly prevalent. Approximately 70% of all children and adolescents have some degree of malocclusion (1–3), and about 35–45% have a more severe malocclusion that needs orthodontic treatment (4). Untreated malocclusions can affect oral health negatively (5). A key part in the evaluation of oral health is the patient-reported outcome measure oral health-related quality of life (OHRQoL) (6). OHRQoL is defined as ‘a multidimensional construct that reflects (among other things) people’s comfort when eating, sleeping and engaging in social interaction; their self-esteem; and their satisfaction with respect to their oral health’ (7). A number of previous systematic reviews have shown that malocclusions in children and adolescents affect OHRQOL negatively (8–11). On the contrary, two more recent systematic reviews concluded that there is a lack of evidence that malocclusions have a negative impact on oral health (12), and that the effect of malocclusions on OHRQoL is inconsistent (13). The influence of malocclusions on OHRQOL may also vary between types of malocclusions (14). Hence, there is still uncertainty about the effects of malocclusions on OHRQoL. Since a significant part of orthodontic treatments is motivated on the basis of improving OHRQoL, it is important to establish whether malocclusions really have a negative impact on OHRQoL. Increased knowledge on the effect of malocclusions on OHRQoL is expected to improve the orthodontic care.

Confounders or confounding variables influence both independent and dependent variables in research, in this case, both malocclusions and OHRQoL. Potential confounders should always be considered when designing studies. Confounders can also be accounted for during statistical analysis, often by multivariate regression models, given that such variables have been measured as a part of the study (15).

Potential confounders in the relationship between malocclusions and OHRQoL are the age and gender of the patient, caries, and socioeconomic status. Previous results on age and OHRQoL are conflicting as it has been shown both that OHRQoL improves between ages 11–12 and 14–15 (16) and that OHRQoL decreases between ages 12 and 15 years (17). The contradictory results may be an indication of the complexity of adolescence. Some studies have shown that girls are more dissatisfied with their teeth than boys (18). Dental caries may result in tooth extractions (19) increasing the risk of developing malocclusions, and untreated caries may per se lead to impaired OHRQoL (20). Furthermore, adolescents with low socioeconomic status have a greater need of orthodontic treatment and poorer OHRQoL (21–25).

Systematic reviews need to be regularly updated. The number of published studies increases annually, leading to a risk that the recent results and previous reviews are inconsistent and out of date (26, 27). The present study is an update of the systematic review published in 2015 by Dimberg *et al.* (11). The previous review presented strong scientific evidence that malocclusions in the aesthetic zone have negative effects on OHRQoL in children and adolescents. However, new published results and potential confounders need to be further evaluated when assessing the scientific evidence concerning malocclusions and OHRQoL in adolescents.

Thus, the aim of this systematic review and meta-analysis is to summarize the results of studies on the impact of malocclusions on OHRQoL in adolescents, after taking potential confounders into consideration.

## Methods

### Definition of the research question

This systematic review and meta-analysis investigate the correlation between malocclusions and OHRQoL in adolescents 10–19 years of age. The following research question is formulated: Do malocclusions in adolescents have a negative impact on OHRQoL when adjusted for potential confounders?

### Protocol and registration

This systematic review follows the Preferred Reporting Items for Systematic Reviews and Meta-Analysis (PRISMA) checklist (28). The protocol was registered on the International prospective register of systematic reviews (PROSPERO) from the National Institute for Health Research database (https://www.crd.york.ac.uk/prospero/) with the protocol no. CRD42020186152.

### Eligibility criteria

The selection criteria for included studies were based on the PECOS strategy (27).

1. **Participants:** Adolescents aged 10–19 years, essentially healthy (ASA class I or II) (29).
2. **Exposure:** Malocclusion and/or orthodontic treatment need rated by professionals using standardized validated measures such as IOTN, DAI, ICON, PAR, or another well-described measurement.
3. **Comparison:** No malocclusions and/or no orthodontic treatment need rated by professionals using standardized measures.
4. **Outcome:** Self-assessed oral health-related quality of life (OHRQoL) using validated instruments.
5. **Study design:** Baseline records of randomized controlled trials (RCTs). Observational studies/non-randomized studies (NRSs): studies of prospective cohorts with an untreated control and cross-sectional studies with a no-malocclusion control. Results from previous systematic reviews could be included, if matching the PECOS.

Exclusion criteria were: (1) severe illness ASA class III or higher, (2) craniofacial syndromes, (3) cleft lip and palate, (4) orthognathic surgery studies, (5) previous or ongoing orthodontic or orthognathic surgery treatment, (6) patients with malocclusion already selected to begin orthodontic treatment, (7) studies only using IOTN-AC for malocclusion rating, (8) studies in languages other than English or Scandinavian languages, and (9) case studies.

### Information sources and search strategy

Five electronic databases (PubMed, Cochrane Library, Cinahl, Scopus, and Web of Science) were searched up to 15 June 2022. The search covers the entire literature and not just studies published since the previous review from 2015, which we aim to update (11). Detailed search strategies of each database are shown in Supplementary Table 1. In addition, PROSPERO and ClinicalTrials.gov were checked for ongoing studies. Finally, the reference lists of the eligible studies were searched for additional studies and a complementary search in Google Scholar was performed on 26 January 2023. Duplicate records were removed using Bramer’s method with the reference management software Endnote (Clarivate Analytics, Philadephia, PA, USA) (30).

### Selection process

Initial screening for eligibility was based on titles and abstracts and performed by all four authors, first independently and then together. If the abstract fulfilled the PECOS, or if it was not possible to assess eligibility from the title and abstract, the full article was obtained. The retrieved full-text articles were imported into the web tool Rayyan software (*https://www.rayyan.ai/*) and split into two sets of articles. Each set contained an equal number of articles and was reviewed for eligibility and assessed by two reviewers working in parallel. Disagreements on inclusion or exclusion were resolved by discussion until achieving consensus among the four reviewers. One of the authors, LD, has previously published articles that were reviewed in this study. LD did not assess her own articles. If more than one article presented data on the same study material/ population, the article with the lowest risk of bias (RoB) or the article presenting baseline values of longitudinal studies was included and the other study was excluded.

### Data extraction and management

The four reviewers discussed and decided which data to extract. One of the authors (EG) performed the data extraction. Accuracy was checked by co-authors.

The following data were extracted from the included studies: (1) authors, publication year, country; (2) study design; (3) study population, age and gender distribution; (4) no malocclusion— rating tool and definition; (5) malocclusion— rating tool and definition; (6) prevalence of malocclusion; (7) quality of life measure; (8) statistical model and confounding variables; (9) effect estimate; and (10) summary of results after adjustment for confounders. The authors of the studies were contacted if data/information was unclear or missing. The extracted data are presented in Table 1.

**Table 1:** Included studies

Study	Study design	Study population, age and gender distribution	No malocclusion—rating tool & definition	Malocclusion—rating tool & definition	Prevalence of malocclusion	Quality of life measure	Statistical model Confounding variables	Effect estimate	Summary of results after adjustment for confounders
Bernabe, 2009, Thailand (43)	Cross-sectional	1034 school children 11–12 years48% females	IOTN DHC 1–3	IOTN DHC 4–5	35%	Generic Child-OIDP & Condition-specific (CS) Child-OIDP	Multiple binary logistic regressionAge, sex, caries, socioeconomics, periodontal disease, traumatic dental injuries	**Odds ratio (95% CI)** Generic Child-OIDP; No malocclusion 1.00 (ref) Malocclusion 0.87 (0.58–1.33) CS Child-OIDP; No malocclusion 1.00 (ref) Malocclusion 2.61 (1.91–3.58)	Malocclusions have no significant impact on OHRQoL measured with generic Child-OIDP. Malocclusions have a negative impact on OHRQoL measured with CS-Child-OIDP.
Bittencourt, 2017, Brazil (41)	Cross-sectional	1612 school children 11–14 years 58% females	DAI ≤ 25	DAI 26–30 definite malocclusion DAI 31–35 severe malocclusion DAI ≥ 36 handicapping malocclusion	31%	CPQ 11-14- ISF:16	Multivariate Poisson regression Age, sex, caries, socioeconomics, traumatic dental injuries	**Prevalence ratio (95% CI)** Normal/minor 1.00 (ref) Definite 1.11 (1.01–1.21) Severe 1.10 (0.98–1.25) Handicapping 1.26 (1.13–1.42)	Malocclusions have a negative impact on OHRQoL. However, for severe malocclusions the difference is not statistically significant.
Dalla Nora, 2022, Brazil (40)	Cross-sectional	1197 school children 15–19 years 57% females	DAI ≤ 25	DAI > 25	76%	OHIP-14	Multilevel Poisson regression Age, sex, caries, socioeconomics, behavioural &, contextual variables	**Rate ratio (95% CI)** No malocclusion 1.00 (ref) Malocclusion 1.26 (1.20–1.32)	Malocclusions have a negative impact on OHRQoL.
Da Rosa, 2016, Brazil (42)	Cross-sectional	1134 school children 12 years 54% females	DAI ≤ 25	DAI 26–30 definite malocclusion DAI 31–35 severe malocclusion DAI ≥ 36 handicapping malocclusion	42%	CPQ 11-14–ISF:16	Multilevel Poisson regression Sex, caries, socioeconomics, traumatic dental injuries	**Rate ratio (95% CI)** Normal/minor 1.00 (ref) Definite 1.07 (1.01–1.12) Severe 1.20 (1.11–1.28) Handicapping 1.26 (1.17–1.35)	Malocclusions have a negative impact on OHRQoL. A dose–response gradient between malocclusion severity and negative impact on OHRQoL was seen.
Dimberg, 2016, Sweden (46)	Cross-sectional, but material derived from a longitudinal study	257 patients from public dental service clinics 9.8–13.5 years 56% females	IOTN DHC grade 1–2	IOTN DHC grade 3–5	43%	CPQ 11-14–ISF:16	Multiple logistic regression Age, sex, socioeconomics Caries did not enter the model (stepwise conditional was applied)	**Odds ratio (95% CI)** No malocclusion 1.00 (ref) Mild 1.40 (0.72–2.72) Borderline 0.61 (0.28–1.32) Severe 1.92 (0.88–4.21) Extreme 0.63 (0.14–2.79)	The effect of malocclusions on OHRQoL is limited an inconsistent.
Feldens, 2016, Brazil (20)	Cross-sectional	509 school children 11–14 years 57% females	DAI ≤ 25	DAI > 25	68%	CPQ 11-14–ISF:16	Multivariate Poisson regression Sex, caries, socioeconomics, traumatic dental injuries	**Rate ratio (95 % CI)** No malocclusion 1.00 (ref) Malocclusion 1.13 (1.00–1.28)	Malocclusions have a negative impact on OHRQoL.
Foster Page, 2012, New Zealand (44)	Cross-sectional	353 school children 12–13 years 48% females	DAI ≤ 25	DAI 26–30 definite malocclusion DAI 31–35 severe malocclusion DAI ≥ 36 handicapping malocclusion	79%	CPQ 11-14–ISF:16	Multivariate regression model Sex, caries, socioeconomics	**Β coefficient (*p*-value)** No malocclusion 0 (ref) Malocclusion 0.092 (0.018)	Malocclusions have a negative impact on OHRQoL.
Kavaliau-skienė, 2020, Lithuania (45)	Cross-sectional	600 school children 15–18 years 59% females	ICON ≤ 43	>43 treatment need	28%	CPQ 11-14, 37 items	Multivariate negative binomial regression (NBR) analysis Age, sex, caries, socioeconomics	**Ratio of sum score means (95% CI)** No malocclusion 1.00 (ref) Malocclusion 1.37 (1.14–1.66)	Malocclusions have a negative impact on OHRQoL.
Mohamed, 2018, Brazil (36)	Cross-sectional	5445 adolescents approached in their home environment 15–19 years 52% females	DAI ≤ 25	DAI 26–30 definite malocclusion DAI 31–35 severe malocclusion DAI ≥ 36 handicapping malocclusion	Not estimable	OIDP	Multivariate Poisson regression Age, sex, caries, socioeconomics, gingivitis	**Β coefficient (SE)** No malocclusion 0 (ref) Malocclusion 0.666 (0.117)	Malocclusions have a negative impact on OHRQoL.
Paula, 2012, Brazil (37)	Cross-sectional	515 school children 12 years 56% females	DAI < 31	DAI ≥ 31	24%	CPQ 11–14, 37 items	Poisson model for multiple regression analysis Sex, socioeconomics, children’s perception of their oral health Caries was not statistically significant in the univariate analysis and was not included in the multivariate model	**Estimative β (SE)** No malocclusion 0 (ref) Malocclusion 0.1183 (0.0382) **Prevalence ratio (p)** No treatment need 1.00 (ref) Treatment need 1.12 (0.0019)	Malocclusions have a negative impact on OHRQoL.
Roque, 2021, Brazil (39)	Cross-sectional	202 school children 11–14 years 54% females	DAI ≤ 25	DAI 26–30 definite malocclusion DAI 31–35 severe malocclusion DAI ≥ 36 handicapping malocclusion	Not estimable	CPQ 11-14–ISF:16	Multiple linear regression Age, sex, caries, socioeconomics, dental trauma	**Coefficient (SE)** No malocclusion 0 (ref) Malocclusion 0.15 (0.07)	Malocclusions have a negative impact on OHRQoL.
Simões, 2017, Brazil (38)	Cross-sectional	417 school children 11–12 years 52% females Part of a larger study presenting 1206 8–12-year-olds	DAI ≤ 25	DAI 26–30 definite malocclusion DAI 31–35 severe malocclusion DAI ≥ 36 handicapping malocclusion	35%	CPQ 11-14–ISF:16	Poisson model for multiple regression analysis Age, sex, caries, socioeconomics, dental trauma	**Adjusted rate ratio (95% CI)** Normal/minor 1.00 (ref) Definite 1.09 (0.90–1.32) Severe 1.17 (0.89–1.54) Very severe 1.28 (1.01–1.62)	Very severe malocclusions have a negative impact on OHRQoL. For definite and severe malocclusions, the differences did not reach statistical significance. A dose–response gradient between malocclusion severity and negative impact on OHRQoL was seen.
Sun, 2017, Hong Kong (35)	Cross-sectional study, baseline values of a longitudinal study	589 school children 12 years 52% females	DAI, ICON, PAR, and IOTN. DAI ratings presented in the article. DAI ratings presented in this table: DAI ≤ 25	DAI 26–30 definite malocclusion DAI 31–35 severe malocclusion DAI ≥ 36 handicapping malocclusion	47%	CPQ 11–14-ISF:8 and CPQ 11–14-RSF:8	Ordinal logistic regression Sex, caries, socioeconomics, periodontal status	**Odds ratio (95 % CI)** CPQ 11–14-ISF:8 Normal/minor 1.00 (ref) Definite 1.35 (0.94–1.94) Severe 1.44 (0.94–2.23) Very severe 1.90 (1.09–3.33) CPQ 11–14-RSF:8 Normal/minor 1.00 (ref) Definite 1.37 (0.95–1.96) Severe 1.59 (1.03–2.45) Very severe 1.64 (0.94–2.87)	When measured with CPQ 11–14-ISF:8, very severe malocclusions have a negative impact on OHRQoL. When measured with CPQ 11–14-RSF:8, severe and very severe malocclusions have a negative impact on OHRQoL. A dose–response gradient between malocclusion severity and negative impact on OHRQoL was seen.

### Study risk of bias (RoB) assessment

To initially calibrate the four reviewers, five articles were assessed for RoB jointly by all four authors. Thereafter, a minimum of two review authors, independently and in duplicate, assessed the RoB in each study using the tool from the SBU developed for evaluating RoB in NRSs (31). The six domains below were considered in included studies, and to qualify for a low risk of bias in each domain the following was required:

1. **Confounding**: Age, gender, caries, and socioeconomic status are considered in an appropriate way, for example, multivariate regression.
2. **Exposure**: Malocclusion rating performed by calibrated professionals under optimal circumstances with access to dental radiographs, for example, in dental clinics.
3. **Drop outs/non participation**: No more than 10% drop outs/non participants.
4. **Measurement of outcome**: Self-assessed OHRQoL using validated instruments.
5. **Selective reporting**: Reasonable outcome measures. Non-significant results were also reported.
6. **Conflict of interest**: Declaration of no conflict of interest.

After assessments of all six domains, an overall RoB classification of ‘low’ (low risk in all domains or some concerns in no more than two domains, none of them being confounding), ‘moderate’ (moderate in two or more domains, none of them being confounding), or ‘high’ (high in confounding or in multiple domains) was made. Studies with a high RoB in the drop out domain could still qualify for an overall judgment of a moderate risk of bias.

Studies not addressing confounders at all were classified as unacceptably high RoB and excluded from further analysis.

Conflicting classifications between the authors were solved by discussion until consensus was achieved.

### Effect measures and synthesis methods

Meta-analysis was performed in studies that presented acceptable homogeneity, identical methods for measuring malocclusions and OHRQoL, and similar effect measures and statistical models. If studies eligible for meta-analysis used the same measurement scales but had sub-grouped malocclusions differently, data for subgroups within studies were pooled to later allow for comparisons between studies (Supplementary Table 4).

In the meta-analysis combining the results of multiple studies, a random-effects model was used to be able to generalize results. Heterogeneity was assessed with the *I*^2^ and Q statistics (32). Meta-analysis was conducted in Stata 17 (StataCorp. 2021. Stata Statistical Software: Release 17. StataCorp LLC, College Station, TX, USA.). Only available data were assessed, that is, missing data were ignored.

### Reporting bias assessment

To minimize the risk of reporting biases, a sensitive search of multiple sources was conducted. If more than 10 studies with similar measures and reporting methods were identified, we planned on conducting a funnel plot and examining it for asymmetry.

### Certainty assessment

Certainty of evidence was assessed using the Grading of Recommendations, Assessments, Development, and Evaluation (GRADE) (33) approach and is presented in Table 2. The level of certainty for each of the comparisons was categorized as high, moderate, low, or very low. The assessment started at ‘high-quality evidence’ (34).

**Table 2:** GRADE summary of findings

OHRQoL in adolescents with malocclusion compared to without malocclusion						
Population: adolescents 10–19 years of age Exposure: malocclusion Comparison: no malocclusion Outcome: OHRQoL						
Outcomes	Nr of studies References Number of study subjects	Study event rates		Relative effect (95% CI)	Quality of evidence according to GRADE	Downgrading
		Without malocclusion	With malocclusion			
Impaired OHRQoL, qualitative synthesis	13 studies (20, 35–46) 13 864	4522/8217^*^ 55%	3695/8217^*^ 45%	Not estimable^**^	⊕⊕⊕⊖^a^ moderate	^a^ Downgraded one level due to indirectness
Impaired OHRQoL, quantitative synthesis (meta-analysis). Malocclusion assessed with DAI and OHRQoL with CPQ 11–14.	4 studies (20, 38, 41, 42) 3672	2199/3672 60%	1473/3672 40%	Rate/prevalence ratio 1.15 (1.12 to 1.18)^***^	⊕⊕⊕⊖^b^ moderate	^b^ Downgraded two levels due to indirectness Upgraded one level due to dose-effect relation

^*^Data on number of people with and without malocclusion not including subjects from Mohamed (36) or Roque (39) studies, as these studies do not present # with and without malocclusion

^**^Not estimable due to differences in measurements of malocclusion, OHRQoL and statistical analysis

^***^Rate ratio (20,38,42), prevalence ratio (41)

**CI:** confidence interval

GRADE Working Group grades of evidence

**High quality:** Further research is very unlikely to change our confidence in the estimate of effect.

**Moderate quality:** Further research is likely to have an important impact on our confidence in the estimate of effect and may change the estimate.

**Low quality:** Further research is very likely to have an impact on our confidence in the estimate of effect and is likely to change the estimate.

**Very low quality:** We are very uncertain about the estimate.

## Results

### Study selection

**Figure 1.**
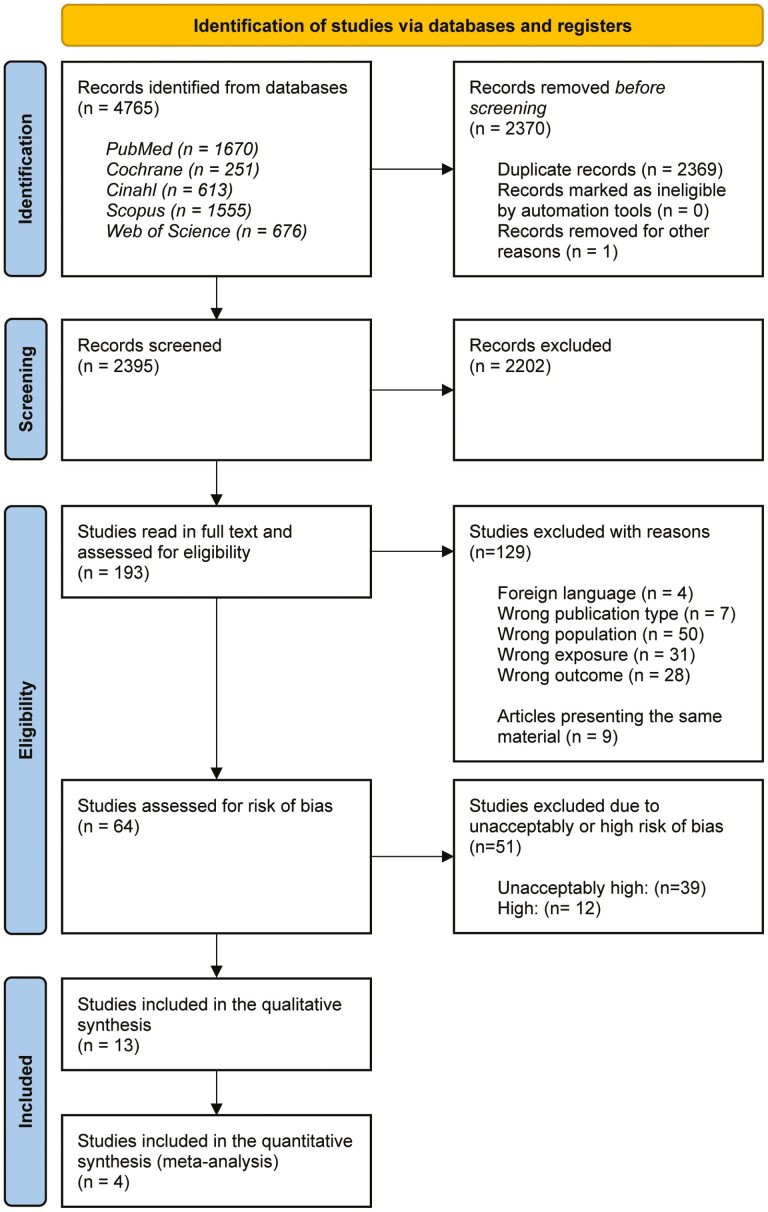
PRISMA flow diagram.

The results of the search are described in a PRISMA flow diagram (28) (Figure 1). Searches were undertaken up to 15 June 2022 and identified 4765 studies. Before screening, 2370 studies were removed, 2369 of these being duplicates and one not being available for abstract assessment. The remaining 2395 titles and abstracts were screened and 2202 studies were excluded. Further on, 193 studies were evaluated in full text and 129 were excluded with a reason (Supplementary Table 2). No additional records were identified from Google Scholar, Prospero, ClinicalTrials.gov, or reference lists. Assessments for risk of bias were made in 64 articles, and 51 articles were excluded due to high or unacceptably high risk of bias (Supplementary Table 3). Finally, 13 studies were found to have a low or moderate risk of bias and were included in the qualitative synthesis. Four of the 13 articles were then also included in the quantitative synthesis (meta-analysis).

### Study characteristics

The 13 included studies involved a total of 13864 patients 10–19 years of age, 7435 girls and 6429 boys. All studies were NRSs of cross-sectional design, although one study presented baseline data of a planned longitudinal design (35). Eight of the studies were conducted in Brazil (20, 36–42). Eight studies were conducted in school settings (20, 37–43), three studies at schools’ dental or medical offices (35, 44, 45), one study at dental clinics (46), and one study in the patients’ home environments (36) (Table 1). The study conducted at dental clinics (46) was the only one mentioning having access to dental radiographs for malocclusion and caries diagnosis.

### Characteristics of malocclusion measurements

The prevalence of malocclusion in included studies ranged between 24% (37) and 79% (44).

The most commonly used rating tool was the Dental Aesthetic Index (DAI), used in 10 of the 13 studies (20, 35–42, 44) (Table 1).

### Characteristics of OHRQoL measurements

Ten studies used the Child Perceptions Questionnaire (CPQ) to assess OHRQoL (20, 35, 37–39, 41, 42, 44–46). Different versions of the CPQ were used: CPQ 11-14-ISF:16 (20, 38, 39, 41, 42, 44, 46), CPQ 11-14-ISF:8 and RSF:8 (35), and the original CPQ 11-14 37 item questionnaire (37, 45). One study used the Generic and Condition–Specific Child Oral Impact on Daily Performances index (Child-OIDP) (43), one the Generic OIDP (36), and one the Oral Health Impact Profile-14 (OHIP-14) (40) (Table 1).

### Reporting bias assessment

As the 13 studies included in the review had different measures, and only 4 of the studies were similar enough to combine in a meta-analysis, we were unable to perform a funnel plot.

### Risk of bias in included studies

**Figure 2.**
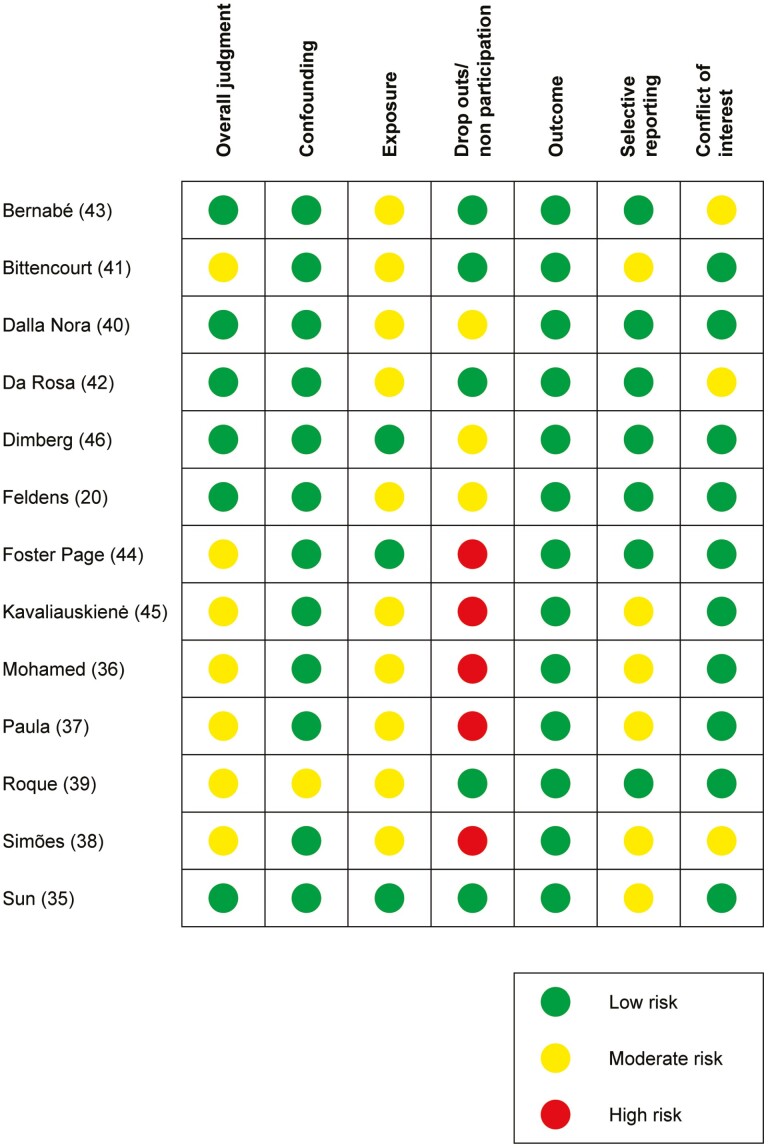
Risk of bias in included studies.

Six articles (20, 35, 40, 42, 43, 46) were assessed at overall low RoB and seven at moderate RoB (36–39, 41, 44, 45). The ratings of overall RoB and for each domain are presented in Figure 2.

### Results of individual studies—impact of malocclusion on OHRQoL when adjusted for confounders

Eight studies reported lower OHRQoL in groups with than without malocclusion (20, 36, 37, 39, 40, 42, 44, 45). In three of four studies comparing malocclusion categories, a dose-effect gradient was found, meaning that OHRQoL gradually decreased when malocclusion increased (35, 38, 42). Three studies found a statistically significant negative impact on OHRQoL for some malocclusion categories but not for all (35, 38, 41). One study reported lower OHRQoL in groups with than without malocclusion using the condition-specific Child-OIDP, but no difference between groups when using the generic Child-OIDP (43). One study concluded that the effect of orthodontic treatment need on OHRQoL was limited and inconsistent (46).

### Results of syntheses

#### Impact of malocclusion on OHRQoL when adjusted for confounders—qualitative synthesis

A majority of the 13 included studies reported lower OHRQoL in groups with than without malocclusions. In three out of four studies comparing malocclusion categories, a dose-effect relation was seen, as OHRQoL gradually decreased when malocclusion increased (Table 1). Since the methods and measures differed, relative and absolute effects were not estimable, and results must be summarized in a narrative way. The conclusion of the qualitative synthesis is that, after adjustment for potential confounding variables, malocclusions have a negative effect on OHRQoL.

#### Impact of malocclusion on OHRQoL when adjusted for confounders—result of quantitative synthesis (meta-analysis)

**Figure 3.**
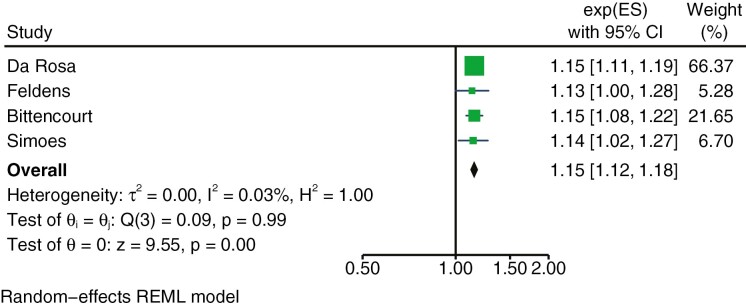
Forest plot of the effect of malocclusions on OHRQoL.

Four articles were included in a quantitative synthesis (meta-analysis) (20, 38, 41, 42). These had the same method of assessing malocclusions (DAI) and OHRQoL (CPQ 11-14-ISF:16). The results of the meta-analysis are that malocclusions in 11- to 14-year-olds measured with DAI have a negative effect on OHRQoL measured with CPQ 11–14 short form (rate ratio/prevalence ratio 1.15 (1.12–1.18) when adjusted for confounders. Heterogeneity was extremely low at *I*^2^ = 0.03% and Q3 = 0.09 (Figure 3).

#### Certainty of evidence

Two outcomes were assessed for certainty of evidence. The first outcome was the qualitative synthesis of whether malocclusions impair OHRQoL. As the methods and measures varied between the 13 included studies, relative and absolute effects were not estimable. We reached moderate quality evidence. Certainty of evidence was downgraded one level due to indirectness because of differences in malocclusion assessments and differences in OHRQoL instruments (34, 47) (Table 2).

The second outcome was more specific about whether malocclusions (measured with DAI) have a negative effect on OHRQoL (measured with CPQ 11–14 ISF 16). The four studies in the quantitative synthesis (meta-analysis) were included in the assessment. We reached moderate quality of evidence that malocclusions lead to impaired OHRQoL when malocclusions are measured with DAI and OHRQoL with CPQ 11–14. Certainty of evidence was downgraded two levels due to indirectness since all four studies were performed in Brazilian school children, which leads to problems with applicability when using these research results to draw conclusions on adolescents worldwide (34, 47). However, certainty of evidence was also upgraded one level due to a dose-effect relation between malocclusion severity and negative OHRQoL (Table 2).

## Discussion

### Main findings

The systematic review and meta-analysis show that malocclusions in adolescents have a negative impact on OHRQoL, after adjustment for potential confounders. The quality of evidence is moderate and based on NRSs. The same conclusion was reached both in the qualitative synthesis with 13 included studies (20, 35–46), and in the quantitative synthesis (meta-analysis) with four included studies (20, 38, 41, 42).

### Study selection

Studies of subjects who had previously received orthodontic treatment were excluded since it is reasonable to believe that untreated individuals without malocclusion are not equivalent to treated individuals without malocclusions (48). To minimize the risk of selection bias, studies with convenience samples, such as patients recruited from orthodontic clinics or dental hospitals, were also excluded. RCTs were included in the PECOS since RCT studies possibly investigating an intervention such as orthodontic treatment could present baseline data of importance.

### Ratings of malocclusion

One shortcoming in the present research field is that the indices used for malocclusion assessments are actually developed to assess treatment need and not malocclusion itself. There is a slight but not insignificant difference between the two. Another problem is that several different treatments need indices and cut-off points are used when rating malocclusions. Four different indices (DAI, IOTN, ICON, and PAR) were used in the included articles (Table 1). Further, studies using the same index sometimes chose different cut-off points for malocclusion. The differences in malocclusion ratings lead to a large spread in the prevalence of malocclusion— ranging between 24% (37) and 79% (44)— and heterogeneity in publication results. It is reasonable to believe that these large differences are explained not only by differences in malocclusion prevalence itself but also by the use of different indices. For example, in a recent study rating individuals using several different indices, prevalence of orthodontic treatment need to be varied between 46% when measured with IOTN (DHC), 57% with DAI, 34% with ICON, and 46% with PAR (49). Perhaps part of the answer to the prevalence discrepancy in the present review could also be inter-examiner disagreements. The research field would be improved if malocclusion assessments were standardized.

It is likely that malocclusion was underdiagnosed in 12 of the 13 included studies since only one study was conducted at dental clinics with access to dental radiographs (46). Ectopic eruption, tooth retention, missing teeth, or supernumerary teeth can hardly be diagnosed without radiographs, especially when assessments are made in the mixed dentition. Malocclusions that are severe but not diagnosed and not visible for the patient most likely do not affect OHRQoL of the individual, which may lead to a risk that the impact of malocclusions on OHRQoL is in fact not as strong as it seems in the presented results. It would perhaps be more correct to state that visible malocclusions have a negative impact on OHRQoL.

### Assessments of OHRQoL

The issue of too many and too varied orthodontic outcome measures has previously been highlighted (50). Research in OHRQoL would be improved if the promising work on Core Outcome Sets (COS) of Orthodontics were adopted worldwide (51). The development of COS is an ongoing work within several subject areas, and guidelines are developed by the international organization COSMIN (52).

Another issue with OHRQoL measurements is that arbitrary cut-off points are often used and that clinically significant effect sizes are lacking (12, 53). In the present systematic review, the most common way of defining a negative effect on OHRQoL was an OHRQoL score above median. However, median values differed between studies, and a score above median is not in itself a perfect indication of poor OHRQoL. Further, it is difficult to say whether a 15% increased risk of an OHRQoL above median for the malocclusion group (the meaning of a rate ratio/prevalence ratio 1.15 (1.12–1.18)) is a large, moderate, or small effect. Research of minimally important differences, the smallest improvements considered worthwhile by the patient, is crucial and should be developed within the OHRQoL field (54).

### Risk of bias assessment

Since all the studies assessed for RoB were observational/NRSs, we used an RoB tool developed for observational studies. We chose the tool developed by and recommended by Swedish Agency for Health Technology Assessment and Assessment of Social Services (HTA).

As it is difficult to measure dropouts in cross-sectional studies, studies with a high risk of bias in the dropout domain could still qualify for an overall judgment of a moderate risk of bias. When evaluating the included studies, the reporting of dropouts varied. In essence, dropouts in cross-sectional studies are individuals declining participation. Some of the included studies reported how many had declined participation, while others did not report on dropouts or declared a 100% participation rate, which is unlikely to be true. To not unfairly exclude studies that actually correctly reported drop outs as the decline of participation, we chose to make this interpretation and assessment.

### Adjustments for confounders

Potential confounders in the relationship between malocclusions and OHRQoL are the age and gender of the patient, caries, and socioeconomic status. In the included studies, age was seldom inserted as a variable in the multivariate analysis. This is reasonable since there was little variation in age within studies. Gender, on the other hand, was considered in all included studies. Caries was most likely underdiagnosed since only one of the included studies used dental radiographs for caries diagnosis. It is beyond our scope to assess the impact of the caries diagnosis on the results in the included studies. Socioeconomic status was the confounder that differed most between the included studies—it was measured in several ways, and the classifications are not comparable between studies.

### Meta-analysis

Four studies were included in the meta-analysis. The authors used CPQ 11–14 ISF 16 for OHRQoL assessments, and the same cut-off point for malocclusion, DAI ≤ 25. However, one study dichotomized the scoring into no malocclusion DAI ≤ 25 and malocclusion DAI > 25 (20). The other three studies (38, 41, 42) subdivided malocclusions into three subgroups: definitive, severe, and very severe malocclusions (Table 1). Ratios for the three malocclusion groups within studies were combined by a fixed-effect model before comparisons between studies could be made (Supplementary Table 4). Without this procedure, a meta-analysis would not have been possible. Of the four studies in the meta-analysis, one presented results as prevalence ratio (41) and three as rate ratio (20, 38, 42). We regarded these ratios as equivalents since they were calculated from the same multivariate Poisson regression analysis (Supplementary Table 4).

### Quality of the evidence

In the early GRADE working days, NRSs always began at the low-quality evidence. However, such a strict grading has later on been questioned. In research fields where RCTs are unethical or unrealistic, NRSs can provide high-quality evidence if potential sources of bias are controlled for (34, 55).

The only feasible study design for the review question is an NRS. It is not possible to randomly assign individuals into developing or not developing malocclusions. Further, it could be unethical to perform an RCT comparing the intervention ‘curing’ malocclusions—orthodontic treatment—to no orthodontic treatment in adolescents. Apart from such a study design being unethical, the orthodontic treatment itself may affect OHRQoL. Probably NRSs are the best types of quantitative research we can hope for with this research question. For these reasons, we began our quality of evidence assessment at ‘high-quality evidence’ (34).

In the quantitative synthesis (meta-analysis) we downgraded due to indirectness since the four studies included in the meta-analysis were all conducted in Brazil. Apparently, Brazilian researchers have a special interest in the effect of malocclusions on OHRQoL. However, due not only to possible cultural differences in how malocclusions affect OHRQoL but also to how the orthodontic care is organized, it would be preferred if studies were more distributed around the world.

### Agreements and disagreements with other studies and reviews

Two systematic reviews on the effect of malocclusions on OHRQoL in children and adolescents have recently been published (12, 13).

In the 2020 review by Macey *et al.*, it was determined that available data could not be included in a meta-analysis (12). The authors further concluded that there is absence of evidence on the effect of malocclusions on OHRQoL, which is a different conclusion than ours. Another difference from our review is that Macey *et al.* did not demand that confounders should be controlled for before inclusion of studies.

The 2021 review by Alrashed and Alqerban did not demand control of confounders for inclusion (13). The review presented two meta-analyses on two subsets of articles: five continuously and six dichotomously analysed studies. The authors concluded that malocclusion has a negative impact on OHRQoL in continuously analysed studies, while in the dichotomously analysed studies malocclusions have a positive impact on OHRQoL. The results are contradictory. In contrast, we only consider four studies similar to be combined in a meta-analysis and our results are more congruent. Another difference when comparing the present review to the study by Alrashed and Alqerban is that our search retrieved approximately four times as many studies despite the fact that the searches were performed at approximately the same point in time. The reason may be that Alrashed and Alqerban only searched MeSH and key terms. Further, the PECOS differed and GRADE was assessed on each study and not on the clinical question/outcome as instructed in the GRADE handbook (47).

When comparing the present review to the systematic review from 2015 (11) that we aim to update, several modifications of the previous protocol have been made. Most importantly, potential confounders had to be controlled for, for example, with a multivariate regression analysis, for studies to be included in our review. Also, the search phrases, the PECOS, the RoB tool, and the GRADE assessment have been updated to current standards. Therefore, the entire literature was searched again, and not only studies published from 2015 and onward. Still, the same conclusion was reached in the current review as in the previous one—that malocclusions have a negative impact on OHRQoL. We can now be more certain of this statement, since the effect was seen even after adjustment for confounders.

### Suggestions for further research

An important part of a systematic review is presenting guidelines on how to conduct an ideal study in the area of interest (56). The following points can improve further NRSs on the effect of malocclusions on OHRQoL;

1. Malocclusion measurements should be standardized. Accepted cut-off points should be used.
2. OHRQoL outcome measures should be standardized and minimal important differences established. Cut-off points for poor OHRQoL should be defined.
3. Confounding variables should be measured and accounted for, for example, in a multivariate regression model.
4. Longitudinal studies of treated and untreated individuals would help us better understand how OHRQoL may differ over time.
5. Future research should also include qualitative studies to improve our understanding of adolescents’ experiences of malocclusions and OHRQoL (57, 58).

## Conclusions

1. This systematic review and meta-analysis confirm earlier findings that malocclusions in adolescents have a negative impact on OHRQoL, after adjustment for potential confounders The quality of evidence is moderate and based on NRSs.
2. More well-designed studies from all over the world would improve our knowledge and the level of evidence about how malocclusions impact children’s and adolescents’ OHRQoL.

## Supplementary Material

Supplementary material is available at the European Journal of Orthodontics online.

cjad009_suppl_Supplementary_Table_S1

cjad009_suppl_Supplementary_Table_S2

cjad009_suppl_Supplementary_Table_S3

cjad009_suppl_Supplementary_Table_S4

## Data Availability

The data underlying this systematic review will be shared on reasonable request to the corresponding author.
