# Supplementary material for: Malocclusions and quality of life among adolescents: a systematic review and meta-analysis
Source: Eur J Orthod. 2023 Mar 30;45(3):295–307. doi: 10.1093/ejo/cjad009 (PMC10230246; doi:10.1093/ejo/cjad009)
Supplement: cjad009_suppl_Supplementary_Table_S1 [file cjad009_suppl_supplementary_table_s1.docx]

| **Supplementary table 1. Search strategy** | |
| --- | --- |
| **Database** | **Keywords** |
| PubMed | "Child"[Mesh] OR "Minors"[Mesh] OR "Adolescent"[Mesh]  OR  “child”[text word] OR “children”[Text word] OR “Child”[Title/Abstract] OR “children”[Title/Abstract] OR “minor*”[Text Word] OR “minor*[Title/Abstract] OR “Adolescent”[Title/Abstract] OR “Adolescence”[Title/Abstract] OR “Youth*”[Title/Abstract] OR “teen*”[Title/Abstract] OR “Young person*”[Title/abstract]  AND  "Malocclusion"[Mesh] OR "Orthodontics"[Mesh] OR "Esthetics, Dental"[Mesh]”  OR  “Malocclusion”[Title/Abstract] OR “Orthodontics”[Title/Abstract] OR “dental esthetic*”[Title/Abstract] OR “dental aesthetic*”[Title/Abstract] AND  "Quality of Life"[Mesh] OR "Self Concept"[Mesh] OR "Patient Satisfaction"[Mesh] OR "Personal Satisfaction"[Mesh]  OR  “Self Concept”[Title/Abstract] OR “Quality of Life”[Title/Abstract] OR “Patient Satisfaction”[Title/Abstract] OR “well being”[Title/Abstract] OR “wellbeing”[Title/Abstract] OR “well being”[Text Word] OR “wellbeing”[Text Word] |
| Cochrane Library | MeSH descriptor: [Child] explode all trees OR MeSH descriptor: [Minors] explode all trees OR MeSH descriptor: [Adolescent] explode all trees OR  (child OR children OR minor* OR Adolescent OR Adolescence OR Youth* OR teen* OR Young person*):ti,ab,kw  AND  MeSH descriptor: [Malocclusion] explode all trees OR MeSH descriptor: [Orthodontics] explode all trees OR MeSH descriptor: [Esthetics, Dental] explode all trees  OR  (Malocclusion OR Orthodontics OR dental esthetic* OR dental aesthetic*):ti,ab,kw  AND  MeSH descriptor: [Quality of Life] explode all trees OR MeSH descriptor: [Self Concept] explode all trees OR MeSH descriptor: [Patient Satisfaction] explode all trees OR MeSH descriptor: [Personal Satisfaction] explode all trees  OR  (Self Concept OR quality of life OR Patient Satisfaction OR well being OR wellbeing):ti,ab,kw |
| Cinahl | (MH "child+") OR (MH "Minors (Legal)") OR (MH "Adolescence+")  OR  TI ( child OR children OR minor* OR Adolescent OR Adolescence OR Youth* OR teen* OR "young person*" )  OR  AB ( child OR children OR minor* OR Adolescent OR Adolescence OR Youth* OR teen* OR "young person*" )  AND  (MH "Malocclusion+") OR (MH "Orthodontics+") OR (MH "Esthetics, Dental")  OR  TI ( Malocclusion OR Orthodontics OR "dental esthetic*" OR "dental aesthetic*" )  OR  AB ( Malocclusion OR Orthodontics OR "dental esthetic*" OR "dental aesthetic*" )  AND MH "Quality of life+" OR MH "Self Concept+" OR MH "Patient Satisfaction+" OR MH "Personal Satisfaction+"  OR  TI ( "Self Concept" OR "quality of life" OR "Patient Satisfaction" OR "well being" OR wellbeing )  OR  AB ( "Self Concept" OR "quality of life" OR "Patient Satisfaction" OR "well being" OR wellbeing ) |
| Scopus | ( TITLE-ABS- KEY ( child  OR  children  OR  minor*  OR  adolescent  OR  adolescence  OR  youth*  OR  teen*  OR  "Young person*" ) )  AND  ( TITLE-ABS-KEY ( malocclusion  OR  orthodontics  OR  "dental esthetic*"  OR  "dental aesthetic*" ) )  AND  ( TITLE-ABS-KEY ( "Self Concept"  OR  "quality of life"  OR  "Patient Satisfaction"  OR  "well being"  OR  wellbeing ) ) |
| Web of Science | ((TS=(((child OR children OR minor* OR adolescent OR adolescence OR youth* OR teen* OR "Young person*") )))  AND  TS=((malocclusion OR orthodontics OR "dental esthetic*" OR "dental aesthetic*")))  AND  TS=(("Self Concept" OR "quality of life" OR "Patient Satisfaction" OR "well being" OR wellbeing) ) |
| Google Scholar | child + malocclusion + quality of life  child + orthodontics + quality of life |
