# Supplementary material for: Malocclusions and quality of life among adolescents: a systematic review and meta-analysis
Source: Eur J Orthod. 2023 Mar 30;45(3):295–307. doi: 10.1093/ejo/cjad009 (PMC10230246; doi:10.1093/ejo/cjad009)
Supplement: cjad009_suppl_Supplementary_Table_S2 [file cjad009_suppl_supplementary_table_s2.docx]

| **Supplementary table 2. Excluded after reading full text** | | |
| --- | --- | --- |
| **Reason for exclusion** | **Studies** | **Number of excluded studies** |
| Foreign language | (1-4) | 4 |
| Wrong publication type | (5-11) | 7 |
| Wrong population | (12-61) | 50 |
| Wrong exposure | (62-92) | 31 |
| Wrong outcome | (93-120) | 28 |
| Studies presenting the same material as another included study | (121-129) | 9 |
| **Total** |  | **129** |

**References**

1. Fernandes, M.L.M.F., Moura, F.M.P., Gamaliel, K.S., and Corrêa-Faria, P. (2013) Dental caries and need of orthodontic treatment: Impact on the quality of life of schoolchildren*.* *Pesquisa Brasileira em Odontopediatria e Clinica Integrada*, 13, 1, 37-43.

2. Filgueira, A.C.G., Machado, F.C.A., Amaral, B.A., Lima, K.C., and Assuncao, I.V. (2016) Oral Health of School´s Teenager*.* *Holos*, 32, 1, 161-172.

3. Oliveira, D.C., Pereira, P.N., Ferreira, F.M., Paiva, S.M., and Fraiz, F.C. (2013) Reported impact of oral alterations on the quality of life of adolescents: A systematic review*.* *Pesquisa Brasileira em Odontopediatria e Clinica Integrada*, 13, 1, 123-129.

4. Chen, X., Liu, F., Li, R., and Wang, Y. (2021) Systematic Review of Factors Influencing Oral Health-related Quality of Life in School-age Children*.* *Chinese General Practice*, 24, 1, 118-124.

5. Hancocks, S. (2011) Summary of: Oral health-related quality of life of children in relation to dental appearance and educational transition*.* *British Dental Journal*, 211, 2, 72-73.

6. Benson, P. (2007) The impact of malocclusion on quality of life*.* *British Dental Journal*, 202, 2, 88-89.

7. Choi, E. and Kim, H.Y. (2016) Oral health related quality of life among Korean adolescents: malocclusion and satisfaction with oral appearance*.* *European Journal of Pediatrics*, 175, 11, 1876-1877.

8. Teodora, J.I., Mihaela, D., and Corneliu, A. (2014) Orthodontic Treatment Need and Quality of Life in Children Living in the Danube Delta Reserve Biosphere*.* *Psychology and Psychiatry, Sociology and Healthcare, Education*, II, 595-605.

9. van Harten, M. (2020) Do socio-economic circumstances affect oral health related quality of life? *Evidence Based Dentistry*, 21, 1, 10-11.

10. Bullying scientifically linked to malocclusion*.* *British Dental Journal*, 211, 12, 587.

11. Agou, S., Locker, D., Tompson, B., and Streiner, D. (2006) Validation of the Child Perceptions Questionnaire in children with malocclusion*.* *Canadian Journal of Dental Hygiene*, 40, 3, 147.

12. Abreu, L.G., Melgaço, C.A., Bastos Lages, E.M., and Paiva, S.M. (2015) Impact of malocclusion on adolescents' oral health-related quality of life*.* *General Dentistry*, 64, 6, e1-e5.

13. Abreu, L.G., Melgaço, C.A., Abreu, M.H., Lages, E.M., and Paiva, S.M. (2015) Agreement between adolescents and parents/caregivers in rating the impact of malocclusion on adolescents' quality of life*.* *Angle Orthodontist*, 85, 5, 806-811.

14. Ahn, Y.S., Kim, H.Y., Hong, S.M., Patton, L.L., Kim, J.H., and Noh, H.J. (2012) Validation of a Korean version of the Child Oral Health Impact Profile (COHIP) among 8- to 15-year-old school children*.* *International Journal of Paediatric Dentistry*, 22, 4, 292-301.

15. Aydoğan, C. (2018) Extraversion and openness to experience moderate the relationship between orthodontic treatment need and oral health-related quality of life in adolescents: (A cross-sectional study)*.* *Angle Orthodontist*, 88, 5, 617-623.

16. Barbosa Tde, S., and Gavião, M.B. (2015) Validation of the Parental-Caregiver Perceptions Questionnaire: agreement between parental and child reports*.* *Journal of Public Health Dentistry*, 75, 4, 255-264.

17. Barbosa Tde, S., Tureli, M.C., Nobre-dos-Santos, M., Puppin-Rontani, R.M., and Gavião, M.B. (2013) The relationship between oral conditions, masticatory performance and oral health-related quality of life in children*.* *Archives of Oral Biology*, 58, 9, 1070-1077.

18. Benson, P.E., Cunningham, S.J., Shah, N., Gilchrist, F., Baker, S.R., Hodges, S.J., and Marshman, Z. (2016) Development of the Malocclusion Impact Questionnaire (MIQ) to measure the oral health-related quality of life of young people with malocclusion: part 2 - cross-sectional validation*.* *Journal of Orthodontics*, 43, 1, 14-23.

19. Benson, P., O'Brien, C., and Marshman, Z. (2010) Agreement between mothers and children with malocclusion in rating children's oral health-related quality of life*.* *American Journal of Orthodontics and Dentofacial Orthopedics*, 137, 5, 631-638.

20. Bernabé, E., de Oliveira, C.M., and Sheiham, A. (2007) Condition-specific sociodental impacts attributed to different anterior occlusal traits in Brazilian adolescents*.* *European Journal Oral Sciences*, 115, 6, 473-478.

21. Brown, A. and Al-Khayal, Z. (2006) Validity and reliability of the Arabic translation of the child oral-health-related quality of life questionnaire (CPQ11-14) in Saudi Arabia*.* *International Journal of Paediatric Dentistry*, 16, 6, 405-411.

22. Caglayan, F., Altun, O., Miloglu, O., Kaya, M.D., and Yilmaz, A.B. (2009) Correlation between oral health-related quality of life (OHQoL) and oral disorders in a Turkish patient population*.* *Medicina Oral, Patologia Oral y Cirugia Bucal*, 14, 11, e573-578.

23. Choi, E.S., Ryu, J.I., Patton, L.L., and Kim, H.Y. (2019) Item-level analysis of the relationship between orthodontic treatment need and oral health-related quality of life in Korean schoolchildren*.* *American Journal of Orthodontics and Dentofacial Orthopedics*, 155, 3, 355-361.

24. Christopherson, E.A., Briskie, D., and Inglehart, M.R. (2009) Preadolescent orthodontic treatment need: objective and subjective provider assessments and patient self-reports*.* *American Journal of Orthodontics and Dentofacial Orthopedics*, 135, 4, S80-86.

25. Christopherson, E.A., Briskie, D., and Inglehart, M.R. (2009) Objective, subjective, and self-assessment of preadolescent orthodontic treatment need--a function of age, gender, and ethnic/racial background? *Journal of Public Health Dentistry*, 69, 1, 9-17.

26. Dawoodbhoy, I., Delgado-Angulo, E.K., and Bernabé, E. (2013) Impact of malocclusion on the quality of life of Saudi children*.* *Angle Orthodontist*, 83, 6, 1043-1048.

27. de Oliveira, C.M., and Sheiham, A. (2003) The relationship between normative orthodontic treatment need and oral health-related quality of life*.* *Community Dentistry and Oral Epidemiology*, 31, 6, 426-436.

28. Fabian, S., Gelbrich, B., Hiemisch, A., Kiess, W., and Hirsch, C. (2018) Impact of overbite and overjet on oral health-related quality of life of children and adolescents*.* *Journal of Orofacial Orthopedics*, 79, 1, 29-38.

29. Gatto, R.C.J., Garbin, A., Corrente, J.E., and Garbin, C.A.S. (2019) The relationship between oral health-related quality of life, the need for orthodontic treatment and bullying, among Brazilian teenagers*.* *Dental Press Journal of Orthodontics*, 24, 2, 73-80.

30. Hope, B., Zaror, C., Sandoval, P., Garay, M., and Streiner, D.L. (2020) Cross-cultural adaptation and validation in spanish of the malocclusion impact questionnaire (MIQ)*.* *Health and Quality of Life Outcomes*, 18, 1, 146.

31. Kavaliauskienė, A., Šidlauskas, A., and Zaborskis, A. (2018) Relationship between orthodontic treatment need and oral health-related quality of life among 11-18-year-old adolescents in Lithuania*.* *International Journal of Environmental Research and Public Health*, 15, 5.

32. Kassis, A., El Osta, N., Tubert-Jeannin, S., Hennequin, M., El Osta, L., and Ghoubril, J. (2018) Cross-cultural adaptation and validation of the child perceptions questionnaire (CPQ11-14) among children in Lebanon*.* *BMC Oral Health*, 18, 1.

33. Klages, U., Erbe, C., Sandru, S.D., Brüllman, D., and Wehrbein, H. (2015) Psychosocial impact of dental aesthetics in adolescence: validity and reliability of a questionnaire across age-groups*.* *Quality of Life Research*, 24, 2, 379-390.

34. Kumar, S., Kroon, J., Lalloo, R., and Johnson, N.W. (2016) Validity and reliability of short forms of parental-caregiver perception and family impact scale in a Telugu speaking population of India*.* *Health and Quality of Life Outcomes*, 14, 34.

35. Li, C., Xia, B., Wang, Y., Guan, X., Yuan, J., and Ge, L. (2014) Translation and psychometric properties of the Chinese (Mandarin) version of the Child Oral Health Impact Profile-Short Form 19 (COHIP-SF 19) for school-age children*.* *Health and Quality of Life Outcomes*, 12, 169.

36. Nagarajappa, R., Batra, M., Sanadhya, S., Daryani, H., and Ramesh, G. (2015) Relationship between oral clinical conditions and daily performances among young adults in India - A cross sectional study*.* *Journal of Epidemiology and Global Health*, 5, 4, 347-357.

37. Oliveira, D.C., Ferreira, F.M., Morosini Ide, A., Torres-Pereira, C.C., Martins Paiva, S., and Fraiz, F.C. (2015) Impact of Oral Health Status on the Oral Health-Related Quality of Life of Brazilian Male Incarcerated Adolescents*.* *Oral Health and Preventive Dentistry*, 13, 5, 417-425.

38. Olivieri, A., Ferro, R., Benacchio, L., Besostri, A., and Stellini, E. (2013) Validity of Italian version of the Child Perceptions Questionnaire (CPQ11-14)*.* *BMC Oral Health*, 13, 55.

39. Pauli, L.A., Correa, M.B., Demarco, F.F., and Goettems, M.L. (2020) The school social environment and oral health-related quality of life in children: a multilevel analysis*.* *European Journal Oral Sciences*, 128, 2, 153-159.

40. Rodd, H.D., Marshman, Z., Porritt, J., Bradbury, J., and Baker, S.R. (2011) Oral health-related quality of life of children in relation to dental appearance and educational transition*.* *British Dental Journal*, 211, 2, E4.

41. Rozan, C., Manso, A.G., Ventura, I., Perez, D.R., Silva, S., and Seiquer, A.C. (2018) Assessment of malocclusion and oral health-related quality of life in Portuguese children using the Child Perceptions Questionnaire (CPQ 11-14)*.* *Annals of Medicine*, 50, S96-S96.

42. Shah, N.D., Arruda, A., and Inglehart, M.R. (2011) Pediatric patients' orthodontic treatment need, quality of life, and smiling patterns -- an analysis of patient, parent, and provider responses*.* *Journal of Public Health Dentistry*, 71, 1, 62-70.

43. Sierwald, I., John, M.T., Sagheri, D., Neuschulz, J., Schüler, E., Splieth, C., Jost-Brinkmann, P.G., and Reissmann, D.R. (2016) The German 19-item version of the Child Oral Health Impact Profile: translation and psychometric properties*.* *Clinical Oral Investigations*, 20, 2, 301-313.

44. Sobouti, F., Kavianpour, Y., Cherati, J.Y., and Dadgar, S. (2020) The Relationship between Malocclusion Severity and Quality of Life in North Iran Students*.* *International Journal of Pediatrics-Mashhad*, 8, 2, 10961-10968.

45. Thiruvenkadam, G., Asokan, S., John, J.B., Geetha Priya, P.R., and Prathiba, J. (2015) Oral health-related quality of life of children seeking orthodontic treatment based on child oral health impact profile: A cross-sectional study*.* *Contemporary Clinical Dentistry*, 6, 3, 396-400.

46. Torres, C.S., Paiva, S.M., Vale, M.P., Pordeus, I.A., Ramos-Jorge, M.L., Oliveira, A.C., and Allison, P.J. (2009) Psychometric properties of the Brazilian version of the Child Perceptions Questionnaire (CPQ11-14) - short forms*.* *Health and Quality of Life Outcomes*, 7, 43.

47. Traebert, E.S., and Peres, M.A. (2007) Do malocclusions affect the individual's oral health-related quality of life? *Oral Health and Preventive Dentistry*, 5, 1, 3-12.

48. Traebert, E.S., and Peres, M.A. (2005) Prevalence of malocclusions and their impact on the quality of life of 18-year-old young male adults of Florianópolis, Brazil*.* *Oral Health and Preventive Dentistry*, 3, 4, 217-224.

49. Wan Hassan, W.N., Yusof, Z.Y., Makhbul, M.Z., Shahidan, S.S., Mohd Ali, S.F., Burhanudin, R., and Gere, M.J. (2017) Validation and reliability of the Malaysian English version of the psychosocial impact of dental aesthetics questionnaire for adolescents*.* *Health and Quality of Life Outcomes*, 15, 1, 54.

50. Wan Hassan, W.N., Yusof, Z.Y., Shahidan, S.S., Mohd Ali, S.F., and Makhbul, M.Z. (2017) Validation and reliability of the translated Malay version of the psychosocial impact of dental aesthetics questionnaire for adolescents*.* *Health and Quality of Life Outcomes*, 15, 1, 23.

51. Zhang, M., McGrath, C., and Hägg, U. (2009) Orthodontic treatment need and oral health-related quality among children*.* *Community Dental Health*, 26, 1, 58-61.

52. Ekuni, D., Furuta, M., Irie, K., Azuma, T., Tomofuji, T., Murakami, T., Yamashiro, T., Ogura, T., and Morita, M. (2011) Relationship between impacts attributed to malocclusion and psychological stress in young Japanese adults*.* *European Journal of Orthodontics*, 33, 5, 558-563.

53. Baskaradoss, J.K., Geevarghese, A., Alsaadi, W., Alemam, H., Alghaihab, A., Almutairi, A.S., and Almthen, A. (2022) The impact of malocclusion on the oral health related quality of life of 11-14-year-old children*.* *BMC Pediatrics*, 22, 1, 91.

54. Iranzo-Cortés, J.E., Montiel-Company, J.M., Bellot-Arcis, C., Almerich-Torres, T., Acevedo-Atala, C., Ortolá-Siscar, J.C., and Almerich-Silla, J.M. (2020) Factors related to the psychological impact of malocclusion in adolescents*.* *Scientific Reports*, 10, 1, 13471.

55. Minamidate, T., Haruyama, N., and Takahashi, I. (2020) The development, validation, and psychometric properties of the Japanese version of the Child Oral Health Impact Profile-Short Form 19 (COHIP-SF 19) for school-age children*.* *Health and Quality of Life Outcomes*, 18, 1, 224.

56. Nguee, A., Ongkosuwito, E.M., Jaddoe, V.W.V., Wolvius, E.B., and Kragt, L. (2020) Impact of orthodontic treatment need and deviant occlusal traits on oral health-related quality of life in children: A cross-sectional study in the Generation R cohort*.* *American Journal of Orthodontics and Dentofacial Orthopedics*, 157, 6, 764-772.

57. Priyadharshini, S., and Leelavathi, L. (2022) Assessment of oral health related quality of life in patients with malocclusion*.* *International Journal of Early Childhood Special Education*, 14, 2, 955-964.

58. Sörensen, C., Lemberger, M., Larsson, P., and Pegelow, M. (2022) Comparing oral health-related quality of life, oral function and orofacial aesthetics among a group of adolescents with and without malocclusions*.* *Acta Odontologica Scandinavica*, 80, 2, 99-104.

59. Zrinski, M.T., Pavlic, A., Katic, V., and Spalj, S. (2022) Effect of personality traits on the association between clinically assessed malocclusion and the psychosocial impact of dental aesthetics*.* *Orthodontics & Craniofacial Research*.

60. Dimberg, L., Arnrup, K., and Bondemark, L. (2015) The impact of malocclusion on the quality of life among children and adolescents: a systematic review of quantitative studies*.* *European Journal of Orthodontics*, 37, 3, 238-247.

61. Liu, Z., McGrath, C., and Hägg, U. (2009) The impact of malocclusion/orthodontic treatment need on the quality of life: a systematic review*.* *Angle Orthodontist*, 79, 3, 585-591.

62. Bhayat, A., and Ali, M.A. (2014) Validity and reliability of the Arabic short version of the child oral health-related quality of life questionnaire (CPQ 11-14) in Medina, Saudi Arabia*.* *Eastern Mediterranean Health Journal*, 20, 8, 477-482.

63. Bianco, A., Fortunato, L., Nobile, C.G., and Pavia, M. (2010) Prevalence and determinants of oral impacts on daily performance: results from a survey among school children in Italy*.* *European Journal of Public Health*, 20, 5, 595-600.

64. Bucci, R., Rongo, R., Zito, E., Valletta, R., Michelotti, A., and D'Anto, V. (2017) Translation and validation of the italian version of the Psychosocial Impact of Dental Aesthetics Questionnaire (pidaq) among adolescents*.* *European Journal of Paediatric Dentistry*, 18, 2, 158-162.

65. Castro Rde, A., Portela, M.C., Leão, A.T., and de Vasconcellos, M.T. (2011) Oral health-related quality of life of 11- and 12-year-old public school children in Rio de Janeiro*.* *Community Dentistry and Oral Epidemiology*, 39, 4, 336-344.

66. da Fonseca, R.C.L., Antunes, J.L.F., Cascaes, A.M., and Bomfim, R.A. (2020) Analysis of the combined risk of oral problems in the oral health-related quality of life of Brazilian adolescents: multilevel approach*.* *Clinical Oral Investigations*, 24, 2, 857-866.

67. Damé-Teixeira, N., Alves, L.S., Ardenghi, T.M., Susin, C., and Maltz, M. (2013) Traumatic dental injury with treatment needs negatively affects the quality of life of Brazilian schoolchildren*.* *International Journal of Paediatric Dentistry*, 23, 4, 266-273.

68. El-Kalla, I.H., Shalan, H.M., and Bakr, R.A. (2017) Impact of Dental Trauma on Quality of Life Among 11-14 Years Schoolchildren*.* *Contemporary Clinical Dentistry*, 8, 4, 538-544.

69. Fakhruddin, K.S., Lawrence, H.P., Kenny, D.J., and Locker, D. (2008) Impact of treated and untreated dental injuries on the quality of life of Ontario school children*.* *Dental Traumatology*, 24, 3, 309-313.

70. Karaman, A.D.D.S.M., and Buyuk, S.K. (2019) Evaluation of temporomandibular disorder symptoms and oral health-related quality of life in adolescent orthodontic patients with different dental malocclusions*.* *Cranio: The Journal of Craniomandibular & Sleep Practice*.

71. Aarabi, G., Reissmann, D.R., Sagheri, D., Neuschulz, J., Heydecke, G., Kofahl, C., and Sierwald, I. (2018) Oral health-related quality of life of children and adolescents with and without migration background in Germany*.* *Quality of Life Research*, 27, 10, 2619-2627.

72. Barakat, L.F. and Ucheonye, I.J. (2016) Oral Health Impact Profile (OHIP-14) and its association with dental treatment needs of adolescents in a rural Nigerian community*.* *Brazilian Journal of Oral Sciences*, 15, 3, 215-220.

73. Kok, Y.V., Mageson, P., Harradine, N.W., and Sprod, A.J. (2004) Comparing a quality of life measure and the Aesthetic Component of the Index of Orthodontic Treatment Need (IOTN) in assessing orthodontic treatment need and concern*.* *Journal of Orthodontics*, 31, 4, 312-318; discussion 300-301.

74. Krisdapong, S., Prasertsom, P., Rattanarangsima, K., and Sheiham, A. (2014) Associations between perceived needs for dental treatment, oral health-related quality of life and oral diseases in school-aged Thai children*.* *Community Dentistry and Oral Epidemiology*, 42, 4, 323-332.

75. Kumar, S., Zimmer-Gembeck, M.J., Kroon, J., Lalloo, R., and Johnson, N.W. (2017) The role of parental rearing practices and family demographics on oral health-related quality of life in children*.* *Quality of Life Research*, 26, 8, 2229-2236.

76. Kumar, S., Kroon, J., Lalloo, R., and Johnson, N.W. (2016) Psychometric Properties of Translation of the Child Perception Questionnaire (CPQ11-14) in Telugu Speaking Indian Children*.* *PLoS One*, 11, 3, e0149181.

77. Locker, D. (2007) Disparities in oral health-related quality of life in a population of Canadian children*.* *Community Dentistry and Oral Epidemiology*, 35, 5, 348-356.

78. Mamani, L., Mercado, S., Mercado, J., and Ríos, K., Jr. (2017) Malocclusion impacts the quality of life of Peruvian school children. A cross-sectional study*.* *Journal of Oral Research*, 6, 9, 234-236.

79. Martins, L.G.T., Pereira, K.C.R., Costa, S.X.S., Traebert, E., Lunardelli, S.E., Lunardelli, A.N., and Traebert, J. (2016) Impact of dental caries on quality of life of school children*.* *Pesquisa Brasileira em Odontopediatria e Clinica Integrada*, 16, 1, 307-312.

80. Naseh, R., Padisar, P., Shojaei-Nejad, H., and Morsaghian, M. (2016) Students' orthodontic treatment needs and oral-health-related quality of life in Qazvin city, Iran*.* *Journal of Oral Health and Oral Epidemiology*, 5, 3, 154-160.

81. Oziegbe, E.O., Esan, T.A., and Adesina, B.A. (2012) Impact of oral conditions on the quality of life of secondary schoolchildren in Nigeria*.* *Journal of Dentistry for Children* 79, 3, 159-164.

82. Peres, K.G., Peres, M.A., Araujo, C.L.P., Menezes, A.M.B., and Hallal, P.C. (2009) Social and dental status along the life course and oral health impacts in adolescents: A population-based birth cohort*.* *Health and Quality of Life Outcomes*, 7.

83. Pulache, J., Abanto, J., Oliveira, L.B., Bönecker, M., and Porras, J.C. (2016) Exploring the association between oral health problems and oral health-related quality of life in Peruvian 11- to 14-year-old children*.* *International Journal of Paediatric Dentistry*, 26, 2, 81-90.

84. Rajab, L.D. and Abu Al Huda, D. (2019) Impact of treated and untreated traumatic dental injuries on oral health-related quality of life among 12-year-old schoolchildren in Amman*.* *Dental Traumatology*, 35, 3, 153-162.

85. Salinas-Martínez, A.M., Hernández-Elizondo, R.T., Núñez-Rocha, G.M., and Ramos Peña, E.G. (2014) Psychometric properties of the Spanish version of the short-form Child Perceptions Questionnaire for 11-14-year-olds for assessing oral health needs of children*.* *Journal of Public Health Dentistry*, 74, 2, 168-174.

86. Thelen, D.S., Bårdsen, A., and Astrøm, A.N. (2011) Applicability of an Albanian version of the OIDP in an adolescent population*.* *International Journal of Paediatric Dentistry*, 21, 4, 289-298.

87. Tubert-Jeannin, S., Pegon-Machat, E., Gremeau-Richard, C., Lecuyer, M.M., and Tsakos, G. (2005) Validation of a French version of the Child-OIDP index*.* *European Journal Oral Sciences*, 113, 5, 355-362.

88. Alsanabani, A.A.M., Yusof, Z.Y.M., Wan Hassan, W.N., Aldhorae, K., and Alyamani, H.A. (2021) Validity and Reliability of the Arabic Version of the Psychosocial Impact of Dental Aesthetics Questionnaire for Yemeni Adolescents*.* *Children (Basel)*, 8, 6.

89. Chaudhary, F.A., Iqbal, A., Khalid, M.D., Noor, N., Syed, J., Baig, M.N., Khattak, O., and Ud Din, S. (2022) Validation and Reliability Testing of the Child Oral Impacts on Daily Performances (C-OIDP): Cross-Cultural Adaptation and Psychometric Properties in Pakistani School-Going Children*.* *Children (Basel)*, 9, 5.

90. Kolawole, K.A. and Ayodele-Oja, M.M. (2020) Oral health-related quality of life of adolescents assessed with the Malocclusion Impact and Child Perceptions questionnaires*.* *American Journal of Orthodontics and Dentofacial Orthopedics*, 159, 2, e149-e156.

91. Lacerda, J.A., Borges, T.M.D., Meneguim, M.C., Vedovello Filho, M., Santamaria Júnior, M., and Vedovello, S.A.S. (2021) Impact of malocclusion severity on the quality of life of non-white adolescents*.* *Cien Saude Colet*, 26, 5233-5240.

92. Benson, P.E., Da'as, T., Johal, A., Mandall, N.A., Williams, A.C., Baker, S.R., and Marshman, Z. (2015) Relationships between dental appearance, self-esteem, socio-economic status, and oral health-related quality of life in UK schoolchildren: a 3-year cohort study*.* *European Journal of Orthodontics*, 37, 5, 481-490.

93. Asgari, I., Ahmady, A.E., Broder, H., Eslamipour, F., and Wilson-Genderson, M. (2013) Assessing the oral health-related quality of life in Iranian adolescents: validity of the Persian version of the Child Oral Health Impact Profile (COHIP)*.* *Oral Health and Preventive Dentistry*, 11, 2, 147-154.

94. Barbosa, T.S., Gavião, M.B., Leme, M.S., and Castelo, P.M. (2016) Oral Health-related Quality of Life in Children and Preadolescents with Caries, Malocclusions or Temporomandibular Disorders*.* *Oral Health and Preventive Dentistry*, 14, 5, 389.

95. Barbosa, T.S., Castelo, P.M., Leme, M.S., and Gavião, M.B. (2012) Associations between oral health-related quality of life and emotional statuses in children and preadolescents*.* *Oral Diseases*, 18, 7, 639-647.

96. Bendo, C.B., Paiva, S.M., Torres, C.S., Oliveira, A.C., Goursand, D., Pordeus, I.A., and Vale, M.P. (2010) Association between treated/untreated traumatic dental injuries and impact on quality of life of Brazilian schoolchildren*.* *Health and Quality of Life Outcomes*, 8, 114.

97. Bernabé, E., Sheiham, A., and de Oliveira, C.M. (2008) Condition-specific impacts on quality of life attributed to malocclusion by adolescents with normal occlusion and Class I, II and III malocclusion*.* *Angle Orthodontist*, 78, 6, 977-982.

98. da Silva, M.F., Vedovello, S.A.S., Vedovello Filho, M., Venezian, G.C., Valdrighi, H.C., and Degan, V.V. (2017) Temporomandibular disorders and quality of life among 12-year-old schoolchildren*.* *Cranio: The Journal of Craniomandibular & Sleep Practice*, 35, 6, 392-396.

99. Paula, J.S., Ambrosano, G.M., and Mialhe, F.L. (2015) Oral Disorders, Socioenvironmental Factors and Subjective Perception Impact on Children's School Performance*.* *Oral Health and Preventive Dentistry*, 13, 3, 219-226.

100. Dos Santos, P.R., Meneghim, M.C., Ambrosano, G.M., Filho, M.V., and Vedovello, S.A. (2017) Influence of quality of life, self-perception, and self-esteem on orthodontic treatment need*.* *American Journal of Orthodontics and Dentofacial Orthopedics*, 151, 1, 143-147.

101. Farzanegan, F., Heravi, F., Sooratgar, A., and Dastmalchi, P. (2014) Evaluation of relationship between oral health-related quality of life and occlusion traits among female adolescents*.* *Dental Research Journal*, 11, 6, 684-688.

102. Gherunpong, S., Tsakos, G., and Sheiham, A. (2006) A socio-dental approach to assessing children's orthodontic needs*.* *European Journal of Orthodontics*, 28, 4, 393-399.

103. Gururatana, O., Baker, S.R., and Robinson, P.G. (2014) Determinants of children's oral-health-related quality of life over time*.* *Community Dentistry and Oral Epidemiology*, 42, 3, 206-215.

104. Heravi, F., Farzanegan, F., Tabatabaee, M., and Sadeghi, M. (2011) Do malocclusions affect the oral health-related quality of life? *Oral Health and Preventive Dentistry*, 9, 3, 229-233.

105. Kragt, L., Jaddoe, V., Wolvius, E., and Ongkosuwito, E. (2017) The association of subjective orthodontic treatment need with oral health-related quality of life*.* *Community Dentistry and Oral Epidemiology*, 45, 4, 365-371.

106. Mbawalla, H.S., Mtaya, M., Masalu, J.R., Brudvik, P., and Astrom, A.N. (2011) Discriminative ability of the generic and condition-specific Child-Oral Impacts on Daily Performances (Child-OIDP) by the Limpopo-Arusha School Health (LASH) project: a cross-sectional study*.* *BMC Pediatrics*, 11, 45.

107. Michel-Crosato, E., et al. (2019) Oral health of 12-year-old children in Quito, Ecuador: a population-based epidemiological survey*.* *BMC Oral Health*, 19, 1, 184.

108. Ramos-Jorge, M.L., Bosco, V.L., Peres, M.A., and Nunes, A.C. (2007) The impact of treatment of dental trauma on the quality of life of adolescents - a case-control study in southern Brazil*.* *Dental Traumatology*, 23, 2, 114-119.

109. Ramos-Jorge, J., Paiva, S.M., Tataounoff, J., Pordeus, I.A., Marques, L.S., and Ramos-Jorge, M.L. (2014) Impact of treated/untreated traumatic dental injuries on quality of life among Brazilian schoolchildren*.* *Dental Traumatology*, 30, 1, 27-31.

110. Torabi-Parizi, M., Karimi-Afshar, M., Mashayekhi, F., Karimi-Afshar, M., and Aminian, A. (2020) Correlation between dental aesthetic index and orthodontics-related quality of life among students in south-east of Iran*.* *Journal of Oral Health and Oral Epidemiology*, 9, 1, 16-23.

111. Tuchtenhagen, S., Bresolin, C.R., Tomazoni, F., da Rosa, G.N., Del Fabro, J.P., Mendes, F.M., Antunes, J.L., and Ardenghi, T.M. (2015) The influence of normative and subjective oral health status on schoolchildren's happiness*.* *BMC Oral Health*, 15, 15.

112. Do, L.G. and Spencer, A. (2007) Oral health-related quality of life of children by dental caries and fluorosis experience*.* *Journal of Public Health Dentistry*, 67, 3, 132-139.

113. Sfreddo, C.S., Moreira, C.H.C., Nicolau, B., Ortiz, F.R., and Ardenghi, T.M. (2019) Socioeconomic inequalities in oral health-related quality of life in adolescents: a cohort study*.* *Quality of Life Research*, 28, 9, 2491-2500.

114. Traebert, J., Lacerda, J.T., Foster Page, L.A., Thomson, W.M., and Bortoluzzi, M.C. (2012) Impact of traumatic dental injuries on the quality of life of schoolchildren*.* *Dental Traumatology*, 28, 6, 423-428.

115. Alvarez-Azaustre, M.P., Greco, R., and Llena, C. (2021) Oral Health-Related Quality of Life in Adolescents as Measured with the Child-OIDP Questionnaire: A Systematic Review*.* *International Journal of Environmental Research and Public Health*, 18, 24.

116. Bourzgui, F., Diouny, S., Mkhantar, H., Serhier, Z., and Othmani, M.B. (2020) Cross-Cultural Adaptation and Validation of "Malocclusion Impact Questionnaire" into Moroccan Arabic*.* *International Dental Journal*, 2020.

117. Comim, L.D., Dalla Nora, Â., Knorst, J.K., Racki, D.N.O., Zenkner, J., and Alves, L.S. (2020) Traumatic dental injury and oral health-related quality of life among 15 to 19 year old adolescents from Santa Maria, Brazil*.* *Dental Traumatology*, 37, 1, 58-64.

118. Jain, V., Agarwal, N., Jabin, Z., Singh, S., Anand, A., and Jain, M. (2020) Cross-cultural adaptation and psychometric properties of the Hindi version of Child Perception Questionnaire (CPQ(11-14) ) in school children*.* *International Journal of Paediatric Dentistry*, 31, 4, 459-467.

119. Macey, R., Thiruvenkatachari, B., O'Brien, K., and Batista, K. (2020) Do malocclusion and orthodontic treatment impact oral health? A systematic review and meta-analysis*.* *American Journal of Orthodontics and Dentofacial Orthopedics*, 157, 6, 738-744.e10.

120. Sun, L., Wong, H.M., and McGrath, C.P.J. (2020) A cohort study of factors that influence oral health-related quality of life from age 12 to 18 in Hong Kong*.* *Health and Quality of Life Outcomes*, 18, 1, 65.

121. Sun, L., Wong, H.M., and McGrath, C.P. (2022) Sociodemographic and Clinical Factors That Influence Oral Health-Related Quality of Life in Adolescents: a Cohort Study*.* *Community Dental Health*, 39, 1, 8-13.

122. Tuchtenhagen, S., Ortiz, F.R., Ardenghi, T.M., and Antunes, J.L.F. (2021) Oral health and happiness in adolescents: A cohort study*.* *Community Dentistry and Oral Epidemiology*, 49, 2, 176-185.

123. Ortiz, F.R., Ardenghi, T.M., Maroneze, M.C., Paiva, S.M., and Pordeus, I.A. (2020) Structuring adolescent's oral health effects on labour market entry in a cohort study*.* *International Journal of Paediatric Dentistry*, 31, 2, 262-269.

124. Ortiz, F.R., Emmanuelli, B., de Campos, A.M., and Ardenghi, T.M. (2022) Oral health-related quality of life determinants throughout adolescence: a cohort study in Brazil*.* *Quality of Life Research*.

125. Machry, R.V., Knorst, J.K., Tomazoni, F., and Ardenghi, T.M. (2018) School environment and individual factors influence oral health related quality of life in Brazilian children*.* *Brazilian Oral Research*, 32, e63.

126. Scapini, A., Feldens, C.A., Ardenghi, T.M., and Kramer, P.F. (2013) Malocclusion impacts adolescents' oral health-related quality of life*.* *Angle Orthodontist*, 83, 3, 512-518.

127. Bendo, C.B., Paiva, S.M., Varni, J.W., and Vale, M.P. (2014) Oral health-related quality of life and traumatic dental injuries in Brazilian adolescents*.* *Community Dentistry and Oral Epidemioly*, 42, 3, 216-223.

128. Sun, L., Wong, H.M., and McGrath, C.P.J. (2018) The factors that influence oral health-related quality of life in 15-year-old children*.* *Health and Quality of Life Outcomes*, 16, 1, 19.

129. Sun, L., Wong, H.M., and McGrath, C.P.J. (2018) The factors that influence oral health-related quality of life in young adults*.* *Health and Quality of Life Outcomes*, 16, 1, 187.
