# Supplementary material for: Malocclusions and quality of life among adolescents: a systematic review and meta-analysis
Source: Eur J Orthod. 2023 Mar 30;45(3):295–307. doi: 10.1093/ejo/cjad009 (PMC10230246; doi:10.1093/ejo/cjad009)
Supplement: cjad009_suppl_Supplementary_Table_S3 [file cjad009_suppl_supplementary_table_s3.docx]

| **Supplementary table 3. Excluded due to high or unacceptably high Risk of Bias** | | | | | | | |
| --- | --- | --- | --- | --- | --- | --- | --- |
|  | **Overall judgment** | **Confounding** | **Exposure** | **Drop outs/ non participants** | **Outcome** | **Selective reporting** | **Conflict of interest** |
| **Excluded due to high RoB** |  | | | | | | |
| Baiju (1) |  |  |  |  |  |  |  |
| Bernabé (2) |  |  |  |  |  |  |  |
| De Paula (3) |  |  |  |  |  |  |  |
| Do (4) |  |  |  |  |  |  |  |
| Elyashkil (5) |  |  |  |  |  |  |  |
| Marques (6) |  |  |  |  |  |  |  |
| O´Brien (7) |  |  |  |  |  |  |  |
| Ravaghi (8) |  |  |  |  |  |  |  |
| Siluvai (9) |  |  |  |  |  |  |  |
| Silva (10) |  |  |  |  |  |  |  |
| Ukra (11) |  |  |  |  |  |  |  |
| Zaborskis (12) |  |  |  |  |  |  |  |
| **Excluded due to unacceptably high RoB (not addressing confounders)** |  | | | | | | |
| (13-51) |  |  | - | - | - | - | - |

|  | Low risk |  | Moderate risk |  | High risk |
| --- | --- | --- | --- | --- | --- |

**References**

1. Baiju, R.M.P., Peter, E., Varghese, N.O., Varughese, J.M., Sivaram, R., and Narayan, V. (2019) Development and initial validation of an oral health-related quality of life scale for older adolescents*.* *Indian Journal of Dental Research*, 30, 6, 826-833.

2. Bernabé, E., de Oliveira, C.M., Sheiham, A., and Tsakos, G. (2009) Comparison of the generic and condition-specific forms of the Oral Impacts on Daily Performances (OIDP) Index*.* *Journal of Public Health Dentistry*, 69, 3, 176-181.

3. Paula, D.F.D.J., Santos, N.C.M., Silva, E.T.D., Nunes, M.D.F., and Leles, C.R. (2009) Psychosocial impact of dental esthetics on quality of life in adolescents*.* *Angle Orthodontist*, 79, 6, 1188-1193.

4. Do, L.G. and Spencer, A. (2007) Oral health-related quality of life of children by dental caries and fluorosis experience*.* *Journal of Public Health Dentistry*, 67, 3, 132-139.

5. Elyaskhil, M., Shafai, N.A.A., and Mokhtar, N. (2021) Effect of malocclusion severity on oral health related quality of life in Malay adolescents*.* *Health and Quality of Life Outcomes*, 19, 1, 71.

6. Marques, L.S., Filogônio, C.A., Filogônio, C.B., Pereira, L.J., Pordeus, I.A., Paiva, S.M., and Ramos-Jorge, M.L. (2009) Aesthetic impact of malocclusion in the daily living of Brazilian adolescents*.* *Journal of Orthodontics*, 36, 3, 152-159.

7. O'Brien, K., Wright, J.L., Conboy, F., Macfarlane, T., and Mandall, N. (2006) The child perception questionnaire is valid for malocclusions in the United Kingdom*.* *American Journal of Orthodontics and Dentofacial Orthopedics*, 129, 4, 536-540.

8. Ravaghi, V., Baker, S.R., Benson, P.E., Marshman, Z., and Morris, A.J. (2019) Socioeconomic variation in the association between malocclusion and oral health related quality of life*.* *Community Dental Health*, 36, 1, 17-21.

9. Siluvai, S., Kshetrimayum, N., Reddy, C.V., Siddanna, S., Manjunath, M., and Rudraswamy, S. (2015) Malocclusion and related quality of life among 13- to 19-year-old students in Mysore City - a cross-sectional study*.* *Oral Health and Preventive Dentistry*, 13, 2, 135-141.

10. Silva, L.F., Thomaz, E.B., Freitas, H.V., Pereira, A.L., Ribeiro, C.C., and Alves, C.M. (2016) Impact of Malocclusion on the Quality of Life of Brazilian Adolescents: A Population-Based Study*.* *PLoS One*, 11, 9, e0162715.

11. Ukra, A., Foster Page, L.A., Thomson, W.M., Farella, M., Tawse Smith, A., and Beck, V. (2013) Impact of malocclusion on quality of life among New Zealand adolescents*.* *New Zealand Dental Journal*, 109, 1, 18-23.

12. Zaborskis, A., Kavaliauskienė, A., and Šidlauskas, A. (2019) Family affluence based inequality in oral health-related quality of life in a population of lithuanian adolescents*.* *International Journal of Environmental Research and Public Health*, 16, 12.

13. Anosike, A.N., Sanu, O.O., and da Costa, O.O. (2011) Malocclusion and its impact on quality of life of school children in Nigeria*.* *West African Journal of Medicine*, 29, 6, 417-424.

14. Anthony, S.N., Zimba, K., and Subramanian, B. (2018) Impact of Malocclusions on the Oral Health-Related Quality of Life of Early Adolescents in Ndola, Zambia*.* *Int J Dent*, 7920973.

15. Araki, M., Yasuda, Y., Ogawa, T., Tumurkhuu, T., Ganburged, G., Bazar, A., Fujiwara, T., and Moriyama, K. (2017) Associations between Malocclusion and Oral Health-Related Quality of Life among Mongolian Adolescents*.* *Int J Environ Res Public Health*, 14, 8.

16. Ashari, A. and Mohamed, A.M. (2016) Relationship of the Dental Aesthetic Index to the oral health-related quality of life*.* *Angle Orthodontist*, 86, 2, 337-342.

17. Barbosa, T.S., Tureli, M.C., and Gavião, M.B. (2009) Validity and reliability of the Child Perceptions Questionnaires applied in Brazilian children*.* *BMC Oral Health*, 9, 13.

18. Bekes, K., John, M.T., Zyriax, R., Schaller, H.G., and Hirsch, C. (2012) The German version of the Child Perceptions Questionnaire (CPQ-G11-14): translation process, reliability, and validity in the general population*.* *Clinical Oral Investigations*, 16, 1, 165-171.

19. Bellot-Arcis, C., Montiel-Company, J.M., and Almerich-Silla, J.M. (2013) Psychosocial impact of malocclusion in Spanish adolescents*.* *Korean Journal of Orthodontics*, 43, 4, 193-200.

20. Bernabé, E., Sheiham, A., and de Oliveira, C.M. (2008) Impacts on daily performances attributed to malocclusions by British adolescents*.* *Journal of Oral Rehabilitation*, 36, 1, 26-31.

21. Bernabé, E., Tsakos, G., de Oliveira, C.M., and Sheiham, A. (2008) Impacts on daily performances attributed to malocclusions using the condition-specific feature of the Oral Impacts on Daily Performances Index*.* *Angle Orthodontist*, 78, 2, 241-247.

22. Bernabé, E., de Oliveira, C.M., and Sheiham, A. (2008) Comparison of the discriminative ability of a generic and a condition-specific OHRQoL measure in adolescents with and without normative need for orthodontic treatment*.* *Health Quality of Life Outcomes*, 6, 64.

23. Bhatia, R., Winnier, J.J., and Mehta, N. (2016) Impact of malocclusion on oral health-related quality of life in 10-14-year-old children of Mumbai, India*.* *Contemporary Clinical Dentistry*, 7, 4, 445-450.

24. Dallé, H., Vedovello, S.A.S., Degan, V.V., De Godoi, A.P.T., Custódio, W., and de Menezes, C.C. (2019) Malocclusion, facial and psychological predictors of quality of life in adolescents*.* *Community Dental Health*, 36, 4, 298-302.

25. Barbosa, T.S., Gavião, M.B., Castelo, P.M., and Leme, M.S. (2016) Factors Associated with Oral Health-related Quality of Life in Children and Preadolescents: A Cross-sectional Study*.* *Oral Health and Preventive Dentistry*, 14, 2, 137-148.

26. Figueroa, F.R., Bancalari, C., Cartes-Velásquez, R., Sanhueza, M., and Palma, C. (2017) Prevalence of malocclusion and its psychosocial impact in a sample of Chilean adolescents aged 14 to 18 years old*.* *Journal of International Dental and Medical Research*, 10, 1, 14-18.

27. Herkrath, F.J., Rebelo, M.A., Herkrath, A.P., and Vettore, M.V. (2013) Comparison of normative methods and the sociodental approach to assessing orthodontic treatment needs in 12-year-old schoolchildren*.* *Oral Health and Preventive Dentistry*, 11, 3, 211-220.

28. Ilijazi-Shahiqi, D., Mehmeti, B., Kelmendi, J., Krasniqi, D., Kamberi, B., and Anic-Milosevic, S. (2020) Validity and reliability of the Psychosocial Impact of Dental Aesthetics Questionnaire in Kosovar adolescents*.* *International Journal of Adolescent Medicine and Health*.

29. Johal, A., Cheung, M.Y., and Marcene, W. (2007) The impact of two different malocclusion traits on quality of life*.* *British Dental Journal*, 202, 2, E2.

30. Kolawole, K.A., Otuyemi, O.D., and Oluwadaisi, A.M. (2011) Assessment of oral health-related quality of life in Nigerian children using the Child Perceptions Questionnaire (CPQ 11-14)*.* *European Journal of Paediatric Dentistry*, 12, 1, 55-59.

31. Kragt, L., Wolvius, E.B., Jaddoe, V.W.V., Tiemeier, H., and Ongkosuwito, E.M. (2018) Influence of self-esteem on perceived orthodontic treatment need and oral health-related quality of life in children: the Generation R Study*.* *European Journal of Orthodontics*, 40, 3, 254-261.

32. Marques, L.S., Ramos-Jorge, M.L., Paiva, S.M., and Pordeus, I.A. (2005) Malocclusion: esthetic impact and quality of life among Brazilian schoolchildren*.* *American Journal of Orthodontics and Dentofacial Orthopedics*, 129, 3, 424-427.

33. Mary, A.V., Mahendra, J., John, J., Moses, J., Ebenezar, A.V.R., and Kesavan, R. (2017) Assessing Quality of Life using the Oral Health Impact Profile (OHIP-14) in Subjects with and without Orthodontic Treatment need in Chennai, Tamil Nadu, India*.* *Journal of Clinical Diagnosic Research*, 11, 8, Zc78-zc81.

34. Masjedi, M., Araban, M., and Arpanah, M. (2019) Relationship between malocclusion and oral health related quality of life among high school girl students in Ahvaz-lran*.* *Medical Science*, 23, 100, 910-919.

35. Montiel-Company, J.M., Bellot-Arcís, C., and Almerich-Silla, J.M. (2013) Validation of the psychosocial impact of dental aesthetics questionnaire (pidaq) in spanish adolescents*.* *Medicina Oral, Patologia Oral y Cirugia Bucal*, 18, 1, e168-e173.

36. Motamedi, M.R.K., Behzadi, A., Khodadad, N., Zadeh, A.K., and Nilchian, F. (2014) Oral health and quality of life in children: A cross-sectional study*.* *Dental Hypotheses*, 5, 2, 53-58.

37. Peter, E., Baiju, R.M., Shivaraman, R., Varghese, N.O., and Varughese, J.M. (2019) Malocclusion-Related Quality of Life Questionnaire (MRQoLQ): Development and validation of a new psychometric tool for older adolescents with malocclusion*.* *Dental Press Journal of Orthodontics*, 24, 6, 28-35.

38. Santos, P.M., Gonçalves, A.R., and Marega, T. (2016) Validity of the Psychosocial Impact of Dental Aesthetics Questionnaire for use on Brazilian adolescents*.* *Dental Press Journal of Orthodontics*, 21, 3, 67-72.

39. Sarit, S., Rajesh, G., Rao, A., Shenoy, R., Pai, M., and Routh, S. (2019) Impact of bruxism on oral health-related quality of life among schoolchildren in Mangaluru city-a case control study*.* *World Journal of Dentistry*, 10, 3, 235-240.

40. Tsakos, G., Gherunpong, S., and Sheiham, A. (2006) Can oral health-related quality of life measures substitute for normative needs assessments in 11 to 12-year-old children? *Journal of Public Health Dentistry*, 66, 4, 263-268.

41. Sun, L., Wong, H.M., and McGrath, C.P.J. (2018) Association Between the Severity of Malocclusion, Assessed by Occlusal Indices, and Oral Health Related Quality of Life: A Systematic Review and Meta-Analysis*.* *Oral Health and Preventive Dentistry*, 16, 3, 211-223.

42. Herkrath, A., Vettore, M.V., de Queiroz, A.C., Alves, P.L.N., Leite, S.D.C., Pereira, J.V., Rebelo, M.A.B., and Herkrath, F.J. (2019) Orthodontic treatment need, self-esteem, and oral health-related quality of life among 12-yr-old schoolchildren*.* *European Journal of Oral Sciences*, 127, 3, 254-260.

43. Traebert, E., Martins, L.G.T., Pereira, K.C.R., Costa, S.X.S., Lunardelli, S.E., Lunardelli, A.N., and Traebert, J. (2018) Malocclusion in Brazilian Schoolchildren: High Prevalence and Low Impact*.* *Oral Health and Preventive Dentistry*, 16, 2, 163-167.

44. de Llano-Pérula, M.C., Ricse, E., Fieuws, S., Willems, G., and Orellana-Valvekens, M.F. (2020) Malocclusion, dental caries and oral health-related quality of life: A comparison between adolescent school children in urban and rural regions in Peru*.* *International Journal of Environmental Research and Public Health*, 17, 6.

45. Foster Page, L.A., Thomson, W.M., Ukra, A., and Baker, S.R. (2013) Clinical status in adolescents: is its impact on oral health-related quality of life influenced by psychological characteristics? *European Journal Oral Sciences*, 121, 3 Pt 1, 182-187.

46. Foster Page, L.A., Thomson, W.M., Jokovic, A., and Locker, D. (2005) Validation of the Child Perceptions Questionnaire (CPQ 11-14)*.* *Journal of Dental Research*, 84, 7, 649-652.

47. Jain, V., Agarwal, N., Jabin, Z., Singh, S., Anand, A., and Jain, M. (2020) Cross-cultural adaptation and psychometric properties of the Hindi version of Child Perception Questionnaire (CPQ(11-14) ) in school children*.* *International Journal of Paediatric Dentistry*, 31, 4, 459-467.

48. Kragt, L., Dhamo, B., Wolvius, E.B., and Ongkosuwito, E.M. (2015) The impact of malocclusions on oral health-related quality of life in children: a systematic review and meta-analysis*.* *Clinical Oral Investigations*, 20, 8, 1881-1894.

49. Ilijazi Shahiqi, D., Dogan, S., Krasniqi, D., Ilijazi, D., and Anic Milosevic, S. (2021) Psycho-social impact of malocclusion in adolescents in Kosovo*.* *Community Dental Health*, 38, 2, 71-75.

50. Foster Page, L.A., Thomson, W.M., Jokovic, A., and Locker, D. (2008) Epidemiological evaluation of short-form versions of the Child Perception Questionnaire*.* *European Journal Oral Sciences*, 116, 6, 538-544.

51. Alrashed, M. and Alqerban, A. (2021) The relationship between malocclusion and oral health-related quality of life among adolescents: a systematic literature review and meta-analysis*.* *European Journal of Orthodontics*, 43, 2, 173-183.
