# Supplementary material for: Malocclusions and quality of life among adolescents: a systematic review and meta-analysis
Source: Eur J Orthod. 2023 Mar 30;45(3):295–307. doi: 10.1093/ejo/cjad009 (PMC10230246; doi:10.1093/ejo/cjad009)
Supplement: cjad009_suppl_Supplementary_Table_S4 [file cjad009_suppl_supplementary_table_s4.docx]

| **Supplementary table 4. Data preparation of studies included in the meta-analysis** | | | | | | | | | | | | | |
| --- | --- | --- | --- | --- | --- | --- | --- | --- | --- | --- | --- | --- | --- |
| **Study** | **No/minor malocclusion**  **RR/PR^b^** | |  | | Definite malocclusion  RR/PR^b^  (95% CI) | | Severe malocclusion  RR/PR^b^  (95% CI) | | Handicapping malocclusion  RR/PR^b^ (95% CI) | |  | **Malocclusion total^a^**  **RR/PR^b^  (95% CI)** | |
| Da Rosa  (42) | 1.00 |  | | 1.07  (1.01, 1.12) | | 1.2  (1.11, 1.28) | | 1.26  (1.17, 1.25) | |  | | 1.15  (1.11, 1.19) |  |
| Feldens  (20) | 1.00 |  | |  | |  | |  | |  | | 1.13  (1.00, 1.28) |  |
| Bittencourt (41) | 1.00 |  | | 1.11  (1.01, 1.21) | | 1.1  (0.98, 1.25) | | 1.26  (1.13, 1.42) | |  | | 1.15  (1.08, 1.22) |  |
| Simões (38) | 1.00 |  | | 1.09  (1.01, 1.32) | | 1.17  (0.98, 1.54) | | 1.28  (1.01, 1.62) | |  | | 1.14  (1.02, 1.27) |  |

^a^ Malocclusion total = definite, severe & handicapping malocclusion subgroups combined.
In study (20) data was presented this way. From studies (42, 41, 38), the three malocclusion groups were pooled/combined into one malocclusion group by meta-analysis fixed effects model to allow for comparisons between studies.
All studies used the DAI index to define malocclusion/no malocclusion.

^b^ Rate ratio/prevalence ratio. One of the studies presented results as prevalence ratio (41), and three as rate ratio (42, 20, 38). These ratios were regarded equivalent since they had been calculated from the same multivariate Poisson regression analysis.
